# Supplementary material for: Transcriptome-wide association study for restless legs syndrome identifies new susceptibility genes
Source: Commun Biol. 2020 Jul 10;3:373. doi: 10.1038/s42003-020-1105-z (PMC7351781; doi:10.1038/s42003-020-1105-z)
Supplement: Supplementary file 1 — Supplementary Information [file 42003_2020_1105_MOESM1_ESM.pdf]

Supplementary Table 1. Number of gene-models for the precomputed expression reference weights.

Tissue	Number of gene models
Amygdala	1837
Anterior cingulate cortex (BA24)	2710
Caudate (basal ganglia)	3661
Cerebellar hemisphere	4408
Cerebellum	5855
Cortex	4012
Frontal cortex (BA9)	3144
Hippocampus	2295
Hypothalamus	2315
Nucleus accumbens (basal ganglia)	3240
Putamen (basal ganglia)	2818
Spinal cord (cervical c-1)	2006
Substantia nigra	1604
Pituitary	4402
Tibial nerve	9657
DLPFC	5420
DLPFC splicing	7772
Total number of tests	67156

Supplementary Table 2. Top TWAS genes for RLS (|Z-score| > 3.50, P < 5E-04). *:Significant after Bonferroni correction

Gene	Tissue	Z-score	P-value	Previous GWAS	Region	Software
DOCK7	Frontal cortex	-3.60	3.23E-04	-	1:62900000	FUSION
S100A3	Cerebellar hemisphere	-3.68	2.29E-04	*PRMT6*	1:106087842	S-PrediXcan
S100A13	Hippocampus	3.62	2.93E-04			S-PrediXcan
S100A16	DLPFC	-3.53	4.20E-04	*PRMT6*	1:153579362	S-PrediXcan
S100A16	Cerebellar hemisphere	-4.03	5.64E-05	*PRMT6*	1:154000000	FUSION
S100A16	DLPFC	-3.62	2.95E-04			FUSION
S100A2	Cerebellum	-3.87	1.07E-04			FUSION
*MEIS1**	DLPFC	-5.40	6.77E-08	*MEIS1*	2:65934026	S-PrediXcan
MEIS1	Putamen basal ganglia	-4.11	3.99E-05	*MEIS1*	2:66700000	FUSION
*MEIS1**	Tibial nerve	6.13	8.88E-10			FUSION
PPP3R1	Tibial nerve	3.67	2.39E-04	*MEIS1*	2:68010206	S-PrediXcan
CCDC148	DLPFC	-3.72	1.98E-04	*CCDC148*	2:158533555	S-PrediXcan
DLX1	DLPFC	-3.75	1.76E-04	-	2:172949468	S-PrediXcan
CMTR1	Caudate basal ganglia	3.87	1.11E-04	*BTBD9*	6:37400995	S-PrediXcan
RP1-153P14.5	Hyptothalamus	-4.06	4.96E-05	*BTBD9*	6:37573165	S-PrediXcan
TAC1	Tibial nerve	-3.55	3.86E-04	-	7:97400000	FUSION
AF131215.2	Cortex	4.67	3.04E-06	-	8:11000000	FUSION
KCTD9	Tibial nerve	3.59	3.32E-04	-	8:25300000	FUSION
GSTO1	DLPFC	3.54	4.03E-04	-	10:105995114	S-PrediXcan
AP000462.3	Cerebellar hemisphere	-3.68	2.33E-04	-	11:115000000	FUSION
CTD-2292M16.8	Cortex	3.54	3.97E-04	-	14:20700000	FUSION
RP5-1021I20.1	Hippocampus	-3.65	2.59E-04	-	14:74300000	FUSION
RP5-1021I20.2	Caudate basal ganglia	-3.52	4.39E-04	-		FUSION
RP5-1021I20.2	Substantia nigra	-3.58	3.46E-04	-		FUSION
IQCH-AS1	Cerebellum	4.15	3.29E-05	*MAP2K5,SKOR1*	15:67094767	S-PrediXcan
AAGAB	DLPFC	-3.59	3.37E-04			S-PrediXcan
RP11-34F13.2	Nerve tibial	-3.74	1.84E-04			S-PrediXcan
*IQCH**	Pituitary	6.37	1.86E-10			S-PrediXcan
IQCH	Nerve tibial	3.55	3.78E-04			S-PrediXcan
IQCH-AS1	Nerve tibial	3.55	3.90E-04			S-PrediXcan
*MAP2K5**	DLPFC	6.40	1.53E-10			S-PrediXcan
*SKOR1**	Pituitary	5.22	1.82E-07			S-PrediXcan
*SKOR1**	Frontal cortex	5.18	2.24E-07			S-PrediXcan
SKOR1	Cerebellum	3.70	2.15E-04			S-PrediXcan

TMEM87A	Cerebellum	3.65	2.60E-04			S-PrediXcan
MAP2K5	Cortex	3.59	3.31E-04	*MAP2K5,SKOR1*	15:67800000	FUSION
MAP2K5	DLPFC	3.72	1.95E-04			FUSION
MAP2K5	Nucleus accumbens basal ganglia	4.76	1.96E-06			FUSION
MAP2K5	Pituitary	3.82	1.35E-04			FUSION
*SKOR1**	Pituitary	5.19	2.14E-07	*MAP2K5,SKOR1*	15:68100000	FUSION
SKOR1	DLPFC	4.65	3.30E-06			FUSION
RP11-645C24.5	Caudate basal ganglia	-3.50	4.68E-04	-	16:21805416	FUSION
*SKAP1**	Caudate basal ganglia	6.02	1.70E-09	-	17:46210802	FUSION
ZGPAT	Caudate basal ganglia	3.68	2.29E-04	*MYT1*	20:62190180	S-PrediXcan
RGS19	Tibial nerve	-3.85	1.17E-04	*MYT1*	20:62704534	FUSION
JAM2	Tibial nerve	3.68	2.31E-04		21:27011584	FUSION

Supplementary Table 3. Splicing events for RLS. *:Significant after Bonferroni correction

**Gene**	**Z-score**	**P-value**	**Previous GWAS**	**Region**
ERC2	-4.20	2.66E-05	-	3:55500000
*SLC36A1**	9.14	6.35E-20	-	5:151000000
PDCD2	4.53	5.91E-06	-	6:171000000
WBSCR22	3.54	4.00E-04	-	7:73100000
MAP2K5	-3.58	3.50E-04	*MAP2K5,SKOR1*	15:67800000
*CCDC57**	5.77	7.84E-09	-	17:80059345
*FN3KRP**	5.94	2.80E-09	-	17:80674581
*NCOA6**	7.55	4.32E-14	-	20:33302577
*NCOA6**	13.25	4.25E-40		
*TRPC4AP**	10.56	4.63E-26	-	20:33590206
YBEY	3.92	8.91E-05	-	21:47706266
S100B	3.66	2.48E-04	-	21:48018530

Supplementary Table 4. Gene-sets and pathways identified using EnrichR

**Gene-set ID**	**Description**	**p-value**
GO:0005509	calcium ion binding	0.0009
GO:2000272	negative regulation of receptor activity	0.0012
GO:0030514	negative regulation of BMP signaling pathway	0.0012
GO:1903845	negative regulation of cellular response to transforming growth factor beta stimulus	0.0032
GO:0090288	negative regulation of cellular response to growth factor stimulus	0.0034
GO:0030510	regulation of BMP signaling pathway	0.0035
GO:0046872	metal ion binding	0.0047
GO:0090101	negative regulation of transmembrane receptor protein serine/threonine kinase signaling pathway	0.0065
R-HSA-380095	Tachykinin receptors bind tachykinins	0.0077

Supplementary Table 5. Druggability analysis

Gene	Drug	CHEMBL_ID	Drug indications	PMIDs
PPP3R1	MYRISTIC ACID	111077	-	10592235
PPP3R1	VOCLOSPORIN	2218919	antiinflammatory agent,DMARD,immunosuppressant	-
UCKL1	CHEMBL214393	214393	-	10592235, 17139284, 17016423
UCKL1	GUANOSINE TRIPHOSPHATE	1233147	-	10592235, 17139284, 17016423
UCKL1	PHOSPHORIBOSYL PYROPHOSPHATE	606077	-	10592235, 17139284, 17016423
UCKL1	URACIL	566	-	10592235, 17139284, 17016423
