# Supplementary material for: Transcriptome-wide association study for restless legs syndrome identifies new susceptibility genes
Source: Commun Biol. 2020 Jul 10;3:373. doi: 10.1038/s42003-020-1105-z (PMC7351781; doi:10.1038/s42003-020-1105-z)
Supplement: Supplementary file 5 — Description of Additional Supplementary Files [file 42003_2020_1105_MOESM5_ESM.pdf]

Description of Additional Supplementary Files

File Name: **Supplementary Data 1**
Description: GWAS traits associated with RLS-TWAS genes

File Name: **Supplementary Data 2**
Description: TWAS traits associated with RLS-TWAS genes

File Name: Supplementary Data 3
Description: **S**ummary statistics of the TWAS and full results of PheWAS
